# Loss of SOX9 Expression Is Associated with PSA Recurrence in ERG-Positive and PTEN Deleted Prostate Cancers

**DOI:** 10.1371/journal.pone.0128525

**Published:** 2015-06-01

**Authors:** Christoph Burdelski, Erzen Bujupi, Maria Christina Tsourlakis, Claudia Hube-Magg, Martina Kluth, Nathaniel Melling, Patrick Lebok, Sarah Minner, Christina Koop, Markus Graefen, Hans Heinzer, Corinna Wittmer, Guido Sauter, Waldemar Wilczak, Ronald Simon, Thorsten Schlomm, Stefan Steurer, Till Krech

**Affiliations:** 1 General, Visceral and Thoracic Surgery Department and Clinic, University Medical Center Hamburg-Eppendorf, Hamburg, Germany; 2 Institute of Pathology, University Medical Center Hamburg-Eppendorf, Hamburg, Germany; 3 Martini-Clinic, Prostate Cancer Center, University Medical Center Hamburg-Eppendorf, Hamburg, Germany; 4 Department of Urology, Section for Translational Prostate Cancer Research, University Medical Center Hamburg-Eppendorf, Hamburg, Germany; 5 Department of Radiology, St. Franziskus-Hospital, Ahlen, Germany; Innsbruck Medical University, AUSTRIA

## Abstract

The transcription factor SOX9 plays a crucial role in normal prostate development and has been suggested to drive prostate carcinogenesis in concert with PTEN inactivation. To evaluate the clinical impact of SOX9 and its relationship with key genomic alterations in prostate cancer, SOX9 expression was analyzed by immunohistochemistry on a tissue microarray containing 11,152 prostate cancers. Data on ERG status and deletions of *PTEN*, 3p13, 5q21 and 6q15 were available from earlier studies. SOX9 expression levels were comparable in luminal cells of normal prostate glands (50% SOX9 positive) and 3,671 cancers lacking TMPRSS2:ERG fusion (55% SOX9 positive), but was markedly increased in 3,116 ERG-fusion positive cancers (81% SOX9 positive, p<0.0001). While no unequivocal changes in the SOX9 expression levels were found in different stages of ERG-negative cancers, a gradual decrease of SOX9 paralleled progression to advanced stage, high Gleason grade, metastatic growth, and presence of PTEN deletions in ERG-positive cancers (p<0.0001 each). SOX9 levels were unrelated to deletions of 3p, 5q, and 6q. Down-regulation of SOX9 expression was particularly strongly associated with PSA recurrence in ERG-positive tumors harboring PTEN deletions (p=0.001), but had no significant effect in ERG-negative cancers or in tumors with normal PTEN copy numbers. In summary, the results of our study argue against a tumor-promoting role of SOX9 in prostate cancer, but demonstrate that loss of SOX9 expression characterizes a particularly aggressive subset of ERG positive cancers harboring PTEN deletions.

## Introduction

Prostate cancer is the most prevalent cancer in men in Western societies [1]. While most tumors have a rather indolent clinical course, prostate cancer still represents the third most common cause of cancer related death in men. Established prognostic parameters are Gleason grade, tumor extent on biopsies, preoperative prostate-specific antigen (PSA), and clinical stage. Although statistically powerful, they are not sufficient for optimal individual treatment decisions. It is hoped that a better understanding of disease biology will eventually lead to the identification of clinically applicable molecular markers that enable a more reliable prediction of prostate cancer aggressiveness in individual patients.

SOX9 belongs to the SOX (SRY-related HMG box) family of developmental transcription factors (reviewed in [2]). It is essential for developmental processes involving sex determination (reviewed in [3]), cartilage development [4], gliogenesis [5], cardiogenesis [6], inner ear formation [7], formation of the hair stem cell compartment [8], progenitor cell pool maintenance in the pancreas [9] and organogenesis of the prostate gland [10]. Here, SOX9 is one of the earliest transcription factors expressed in the primordial prostate [10]. In the adult prostate, SOX9 expression is strongly expressed in the basal cells [11,12], where it has an important role for maintenance of normal prostate function [11,13]. Several lines of evidence exist that SOX9 might also contribute to prostate cancer initiation and progression, including up-regulation during early stages of prostate neoplasia in mouse models [14], as well as in human prostatic intraepithelial neoplasia (PIN) [10] and prostate cancers [10,11] [14–17]. Some of these studies on 98–387 patients have also linked SOX9 overexpression to high-grade and advanced tumors [10,14,15,17], hormone-refractory disease state [15] or poor patient prognosis [14,15,17].

The promising findings of these studies obtained in limited patient sets prompted us to further evaluate the possible clinical impact of SOX9 in prostate cancer. For this purpose, we took advantage of our preexisting tissue microarray containing >11,000 prostate cancer specimens with clinical follow-up and attached molecular database. The results of our study demonstrate that decreased, and not elevated, SOX9 protein expression is linked to poor prognosis and that this effect is strictly limited to the subset of prostate cancers harboring PTEN deletions.

## Materials and Methods

### Patients

Radical prostatectomy specimens were available from 11,152 patients, undergoing surgery between 1992 and 2011 at the Department of Urology and the Martini Clinics at the University Medical Center Hamburg-Eppendorf. Follow-up data were available for a total of 9,695 patients with a median follow-up of 31.2 months (range: 0.3 to 228 months; Table 1). Gleason grading was performed according to criteria summarized in the 2004 World Health Organization (WHO) classification of genito-urinary cancers [18]. Prostate specific antigen (PSA) values were measured following surgery and PSA recurrence was defined as the time point when postoperative PSA was at least 0.2 ng/ml and increasing at subsequent measurements. All prostate specimens were analyzed according to a standard procedure, including a complete embedding of the entire prostate for histological analysis [19]. The TMA manufacturing process was described earlier in detail [20]. In short, one 0.6 mm core was taken from a representative tissue block from each patient. The tissues were distributed among 24 TMA blocks, each containing 144 to 522 tumor samples. For internal controls, each TMA block also contained various control tissues, including normal prostate tissue. The molecular database attached to this TMA contained results on ERG expression in 9,619 [21], *ERG* break apart FISH analysis in 6,106 (expanded from [21]) and deletion status of 5q21 (*CHD1)* in 7,222 (expanded from [22]), 6q15 (*MAP3K7)* in 3,523 (expanded from [23]), *PTEN* (10q23) in 6,109 (expanded from [24]) and 3p13 (*FOXP1)* in 6,410 (expanded from [25]) cancers.

**Table 1:** Composition of the prognosis tissue microarray containing 11,152 prostate cancer specimens.

	No. of patients	
	Study cohort on tissue microarray (n = 11,152)	Biochemical relapse among categories (n = 1,824)
**Follow-up (mo)**		
Mean	53.4	-
Median	36.8	-
**Age (y)**		
<50	318	49
50–60	2,768	460
60–70	6,548	1,081
>70	1,439	232
**Pretreatment PSA (ng/ml)**		
<4	1,407	142
4–10	6,735	827
>10–20	2,159	521
>20	720	309
**pT category (AJCC 2002)**		
pT2	7,370	570
pT3a	2,409	587
pT3b	1,262	618
pT4	63	49
**Gleason grade**		
≤3+3	2,859	193
3+4	6,183	849
4+3	1,565	573
≥4+4	482	208
**pN category**		
pN0	6,117	1,126
pN+	561	291
**Surgical margin**		
negative	8,984	1,146
positive	1,970	642

### Ethics statement

The usage of archived diagnostic left-over tissues for manufacturing of tissue microarrays and their analysis for research purposes as well as patient data analysis has been approved by local laws (HmbKHG, §12,1) and by the local ethics committee (Ethics commission Ärztekammer Hamburg, WF-049/09 and PV3652). According to local laws, informed consent was not required for this study. Patient records/information was anonymized and de-identified prior to analysis. All work has been carried out in compliance with the Helsinki Declaration.

### Immunohistochemistry

Freshly cut TMA sections were immunostained on one day and in one experiment. Slides were de-waxed and exposed to heat-induced antigen retrieval for 5 minutes in an autoclave at 121°C in pH 9 Dako Target Retrieval Solution. Primary antibody specific for SOX9 (mouse monoclonal antibody (clone 3C10), Abnova, Taipei City, Taiwan; cat# H00006662-M02; dilution 1:700) was applied at 37°C for 60 minutes. Bound antibody was then visualized using the EnVision Kit (Dako, Glostrup, Denmark) according to the manufacturer´s directions. If present, SOX9 staining was typically homogenous in the nuclei of all cancer cells of the tissue spots. The percentage of positive tumor cells (typically 100%) was thus not recorded separately. Faint cytoplasmic staining, which accompanied nuclear staining in most tissue spots, was attributed to non-specific staining and not considered for the analysis. Nuclear staining intensity of all cases was semiquantitatively assessed in four categories: negative, weak, moderate and strong.

### Statistics

Statistical calculations were performed with JMP 9 software (SAS Institute Inc., NC, USA). Contingency tables and the chi^2^-test were applied to search for associations between molecular parameters and tumor phenotype. Survival curves were calculated according to Kaplan-Meier. The Log-Rank test was applied to detect significant differences between groups. Cox proportional hazards regression analysis was performed to test the statistical independence and significance between pathological, molecular and clinical variables.

## Results

### Technical aspects

A total of 3,587 of 11,152 (32%) tissue samples were non-informative. Three TMA sections comprising 1,271 samples were excluded from analysis because of insufficient staining quality. These three sections have not been re-stained in order to avoid a staining intensity bias that might occur when IHC experiments are repeated on different days. In another 2,316 tissue spots, no IHC result was obtained due to the complete lack of tissue or absence of unequivocal cancer cells.

### SOX9 in prostate cancer

**Fig 1:**
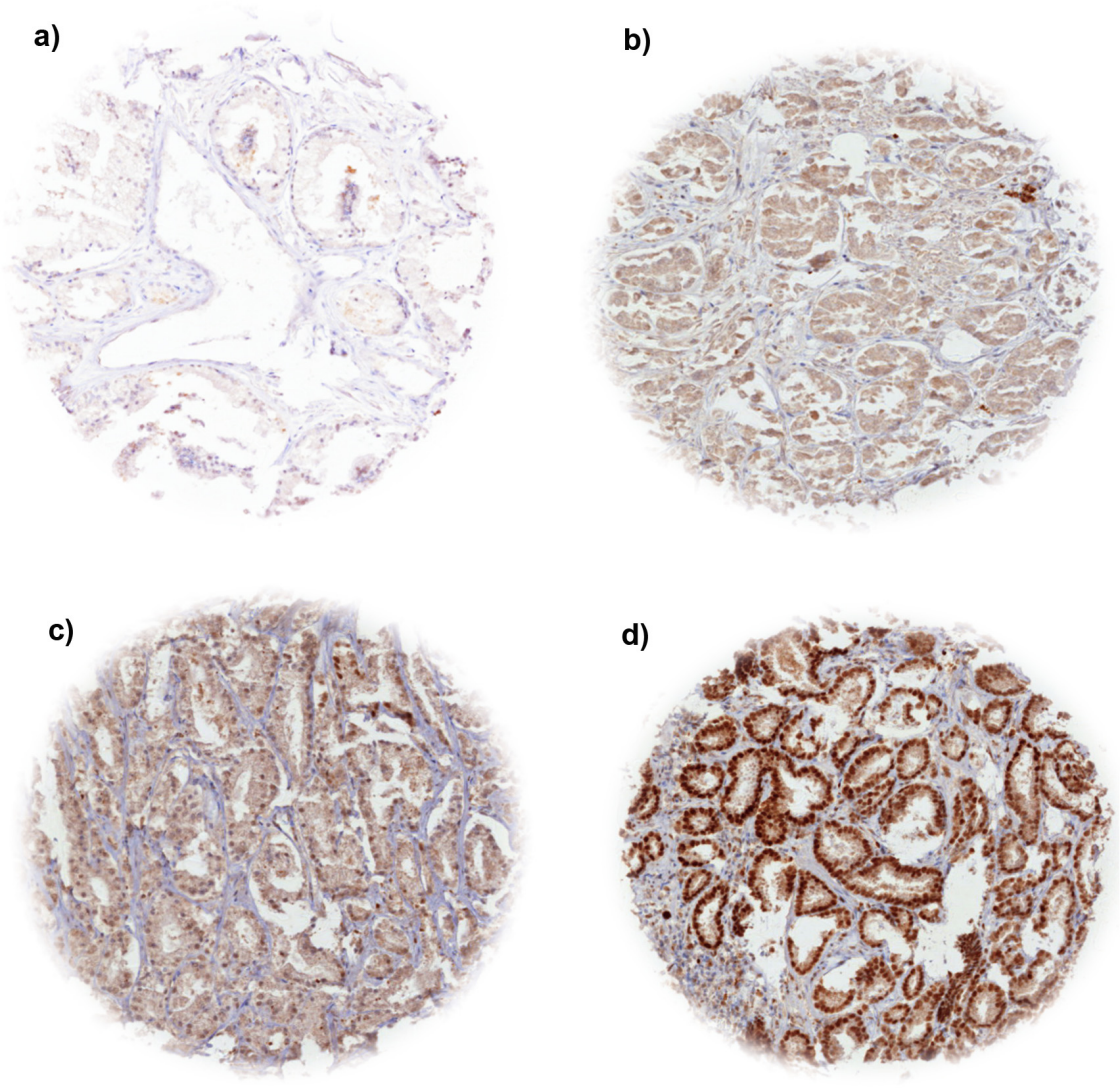
Representative pictures SOX9 immunostaining in prostate cancer. (a) negative, (b) weak, (c) moderate, (d) strong.

Nuclear SOX9 expression was typically strong in basal cells of normal prostate glands. In normal luminal cells, moderate to strong staining was found in about 50% of analyzed cases. In prostate cancer, nuclear SOX9 expression was observed in 67% of 7,565 interpretable cases. Detectable SOX9 staining was considered weak in 15%, moderate in 39% and strong in 13% of cases. Examples of tissue spots with variable levels of SOX9 immunostaining are shown in Fig 1.

### Association with ERG status

**Fig 2:**
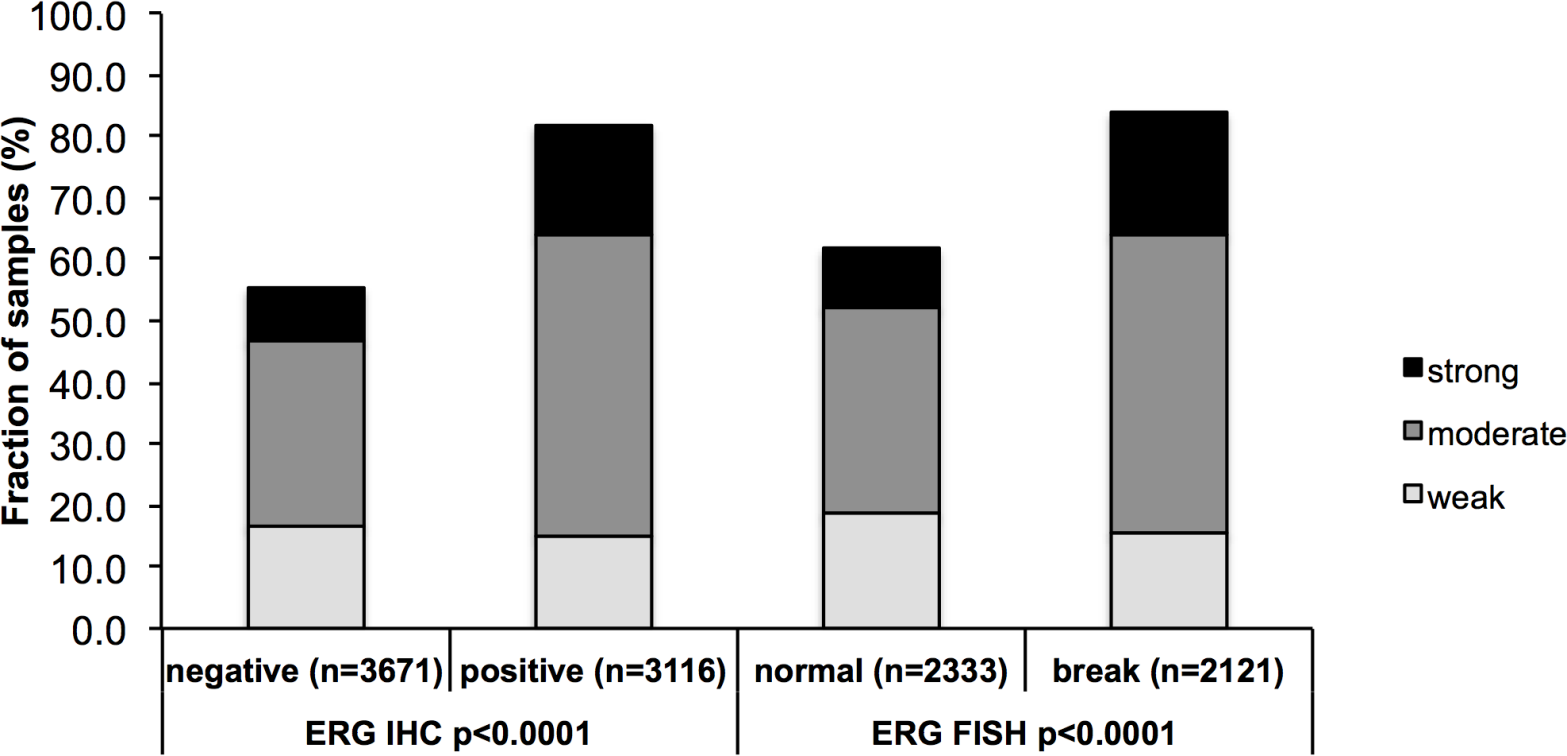
Relationship of SOX9 expression with ERG status. IHC = immunohistochemistry; FISH = fluorescence in-situ hybridization.

Data on both interpretable SOX9 staining and ERG status were available from 7,565 tumors if the ERG status was determined by IHC analysis and from 4,454 tumors if ERG rearrangement was analysed by FISH. SOX9 staining was strongly linked to ERG-positive cancers irrespective of whether the ERG status was analyzed by IHC or FISH: SOX9 expression was found in 82% of ERG IHC-positive and 83% of ERG FISH-rearranged cancers, but only in 55% of ERG IHC-negative and 62% of ERG FISH-normal cancers (p<0.0001 each, Fig 2).

### Association with other key genomic alterations

**Fig 3:**
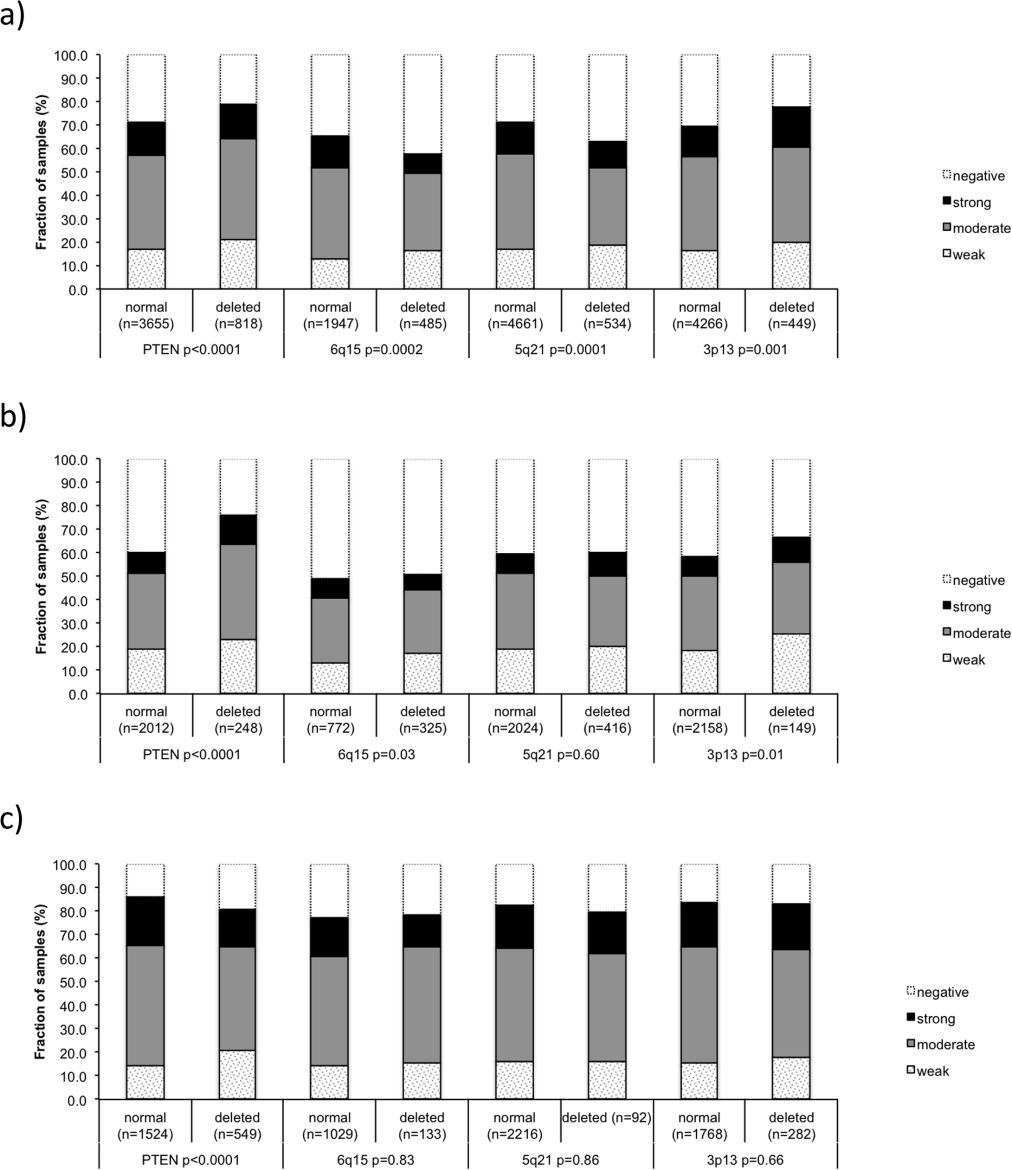
SOX9 expression versus *PTEN*, 3p13, 6q15 and 5q21 deletions probed by FISH analysis. (a) all cancers, (b) in ERG-negative, c) ERG-positive subset.

Earlier studies have provided evidence for distinct and clinically relevant molecular subgroups of prostate cancers defined by gene rearrangements and several genomic deletions. Others and us described a strong link between PTEN, 3p13 deletions and ERG positivity, and between 5q21, 6q15 deletions and ERG negativity [22–24,26–28]. To study whether SOX9 expression might be linked to any of these alterations, SOX9 data were compared with preexisting findings on deletions of PTEN, 3p13, 6q15 and 5q21. In the analysis of all tumors, SOX9 staining was positively associated with deletions of PTEN (p<0.0001) and 3p13 (p = 0.001) and negatively associated with deletions of 6q15 (p = 0.0002) and 5q21 (p<0.0001, Fig 3A). This was expected because all these deletions—as like SOX9—are known to be tightly linked to the ERG status. Indeed, when stratified for subsets of ERG-fusion positive and negative cancers, most of the association disappeared. However, for PTEN, the separate analysis of ERG-positive and ERG-negative cancers revealed a striking bimodal relationship with SOX9 expression. PTEN deletions were positively associated with SOX9 expression in ERG-negative (p<0.0001, Fig 3B) but inversely linked to SOX9 expression in ERG-positive cancers (p<0.0001, Fig 3C).

### Relationship with tumor phenotype

Reduced SOX9 expression was linked to an unfavorable tumor phenotype, including late stage, high Gleason grade, and elevated preoperative PSA levels (p<0.0001 each, Table 2) in all cancers. However, separate analyses of ERG-positive and ERG-negative cancers revealed that this association was solely driven by the subset of ERG-positive cancers. Here, reduced SOX9 expression was strongly linked to advanced tumor stage, high Gleason grade, high preoperative PSA levels, presence of nodal metastases (p<0.0001 each) and positive surgical margin (p = 0.001) (Table 3). In contrast, no unequivocal differences were found between SOX9 levels and tumor phenotype in ERG-negative cancers, although significant p-values were still obtained due to the very high number of samples in our study (S1 Table).

**Table 2:** Clinico-pathological association of SOX9 immunostaining in all cancers.

Parameter	Evaluable (n)	SOX9 (%)				P value
		negative	weak	moderate	strong	
**Total**	7565	33	15	39	13	
**Tumor stage**						<0.0001
pT2	4921	32	14	40	15	
pT3a	1725	34	18	37	11	
pT3b	848	34	20	36	10	
pT4	40	43	25	28	5	
**Gleason grade**						<0.0001
≤3+3	1741	38	11	37	14	
3+4	4363	30	16	40	14	
4+3	1115	34	20	36	10	
≥4+4	306	38	19	32	11	
**Lymph node metastasis**						0.04
N0	4233	32	17	39	13	
N+	371	36	18	37	9	
**Preoperative PSA level (ng/ml)**						<0.0001
<4	905	25	14	43	18	
4–10	4597	31	15	41	14	
>10–20	1487	37	18	33	12	
>20	491	48	17	29	6	
**Surgical margin**						0.01
negative	6053	32	15	39	14	
positive	1374	37	16	36	12	

**Table 3:** Clinico-pathological association of SOX9 immunostaining in the ERG positive subset.

Parameter	Evaluable (n)	SOX9 (%)				P value
		negative	weak	moderate	strong	
**Total**	3116	18	15	49	18	
**Tumor stage**						<0.0001
pT2	1898	15	12	52	21	
pT3a	839	20	19	47	14	
pT3b	352	30	21	36	14	
pT4	15	33	33	33	0	
**Gleason grade**						<0.0001
≤3+3	695	21	10	50	19	
3+4	1879	16	16	50	19	
4+3	433	23	22	42	14	
≥4+4	93	30	19	40	11	
**Lymph node metastasis**						<0.0001
N0	1757	18	17	48	18	
N+	156	31	19	42	8	
**Preoperative PSA level (ng/ml)**						<0.0001
<4	428	16	13	50	22	
4–10	1924	16	14	52	18	
>10–20	546	21	19	41	19	
>20	181	32	21	39	8	
**Surgical margin**						0.001
negative	2466	17	15	50	19	
positive	593	24	16	45	16	

### Relationship with PSA recurrence

**Fig 4:**
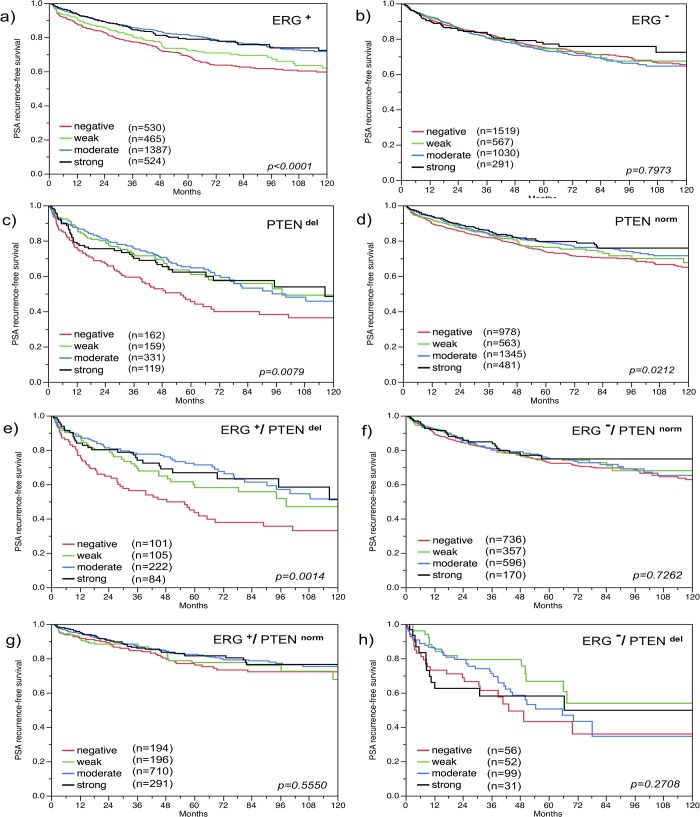
Association of SOX9 expression with biochemical recurrence. (a) ERG-positive (ERG^**+**^) cancers (n = 2,906), (b) ERG-negative (ERG^**-**^) cancers (n = 3,407), (c) *PTEN* deleted (PTEN^**del**^) cancers (n = 771), (d) *PTEN* non-deleted (PTEN^**norm**^) cancers (n = 3,367), (e) ERG-positive and *PTEN* deleted cancers (n = 512), (f) ERG-negative and *PTEN* non-deleted cancers (n = 1,859), (g) ERG-positive and *PTEN* non-deleted cancers (1,391), (h) ERG-negative and *PTEN* deleted cancers (n = 238). A detailed list of the number of patients at risk is given for (a) and (b) in S3 Table.

The prognostic value of SOX9 expression depended on the ERG status. Absent or reduced SOX9 expression was strongly linked to biochemical (PSA) recurrence in the subset of ERG-positive cancers (p<0.0001, Fig 4A), but not in ERG-negative cancers (p = 0.80, Fig 4B). Because SOX9 expression was linked to the PTEN status, we also compared the prognostic impact of SOX9 in cancers with and without PTEN deletions. This analysis revealed, that a loss of SOX9 expression was strongly linked to PSA recurrence in PTEN-deleted cancers (p = 0.008, Fig 4C) but only weakly in cancers with normal PTEN copy numbers (p = 0.02, Fig 4D). The strongest association between loss of SOX9 and poor outcome was found in the subset of cancers harboring both ERG-fusion and PTEN deletion (p = 0.001, Fig 4E), while SOX9 was unrelated to prognosis in ERG-positive and ERG-negative cancers with normal PTEN status (p = 0.56 and p = 0.73; Fig 4G and 4F) or in ERG-negative cancers with PTEN deletion (p = 0.27; Fig 4H).

### Multivariate analysis

In order to estimate whether the clinical impact of SOX9 expression in ERG-positive cancers harboring PTEN deletions was independent from established prognostic parameters, we performed four different types of multivariate analyses Scenario 1 evaluated all postoperatively available parameters including pathological tumor stage, pathological lymph node status (pN), surgical margin status, preoperative PSA value and pathological Gleason grade obtained after the morphological evaluation of the entire resected prostate. In scenario 2, all postoperatively available parameters with exception of nodal status were included. The rational for this approach was that the indication and extent of lymph node dissection is not standardized in the surgical therapy of prostate cancer and that excluding pN in multivariate analysis can markedly increase case numbers. Two additional scenarios had the purpose to model the preoperative situation as much as possible. Scenario 3 included SOX9 expression, preoperative PSA, clinical tumor stage (cT stage) and Gleason grade obtained on the prostatectomy specimen. Since postoperative determination of a tumor’s Gleason grade is “better” than the preoperatively determined Gleason grade (subjected to sampling errors and consequently under-grading in more than one third of cases [29]), another multivariate analysis was added. In scenario 4, the preoperative Gleason grade obtained on the original biopsy was combined with preoperative PSA, cT stage and SOX9 expression. This analysis, which was limited to the subset of ERG-positive and PTEN-deleted cancers where SOX9 had strongest prognostic impact in univariate analysis, revealed that the prognostic value of SOX9 was not independent from the established prognosticators (S2 Table).

## Discussion

The results of our study show that a clinical relevance of SOX9 expression levels exists in prostate cancer that greatly depends on the molecular context of the tumor cells. In particular, our data demonstrate that the prognostic relevance of SOX9 expression is strictly limited to the subset of ERG-positive tumors harboring PTEN deletions.

The fraction of cancers with detectable SOX9 expression in this study was 67%, including 51% with moderate to strong staining. These numbers are in the upper range of earlier immunohistochemistry studies reporting 41–55% SOX9 positive cancers by conventional large section analysis involving up to 36 tumors [11,16], or of TMA studies reporting 46–100% SOX9 positivity in up to 387 prostate cancers [10,14,17]. It is most likely that these differences are first of all related to technical issues, including usage of different antibodies and scoring criteria. That comparable fractions of SOX9 positive cancers can be found with both large section and TMA approaches suggests that our analysis provided representative data, which are not markedly influenced by sampling error issues that can potentially occur in studies evaluating small tissue cores measuring only 0.6 mm in diameter per patient.

The successful analysis of more than 7,500 prostate cancers did not—in a general survey on all tumors—reveal relevant associations of an increased SOX9 expression with unfavorable tumor phenotype or poor patient prognosis as suggested by earlier works [10,14,15,17]. Multiple studies involving 98–387 cancers had previously suggested a link between SOX9 expression and poor tumor features, such as high Gleason grade [10,14,15,17] and even shortened overall patient survival [14]. In contrast, our data revealed a better patient outcome in case of high SOX9 expression levels in subgroups. In line with our findings, Zhong et al. demonstrate a prolongued recurrence-free interval for tumors with elevated SOX9 levels in a cohort of 147 patients [15].

The molecular database attached to our prostate cancer TMA enabled an evaluation of the relationship between SOX9 expression and other key molecular features of prostate cancer. About 50% of prostate cancers are characterized by the TMPRSS2:ERG fusion, which results in overexpression of the ERG transcription factor and massive transcriptional changes [30]. Since ERG regulates the androgen-receptor (AR) responsive SOX9 indirectly by functioning as a pioneer factor to open a cryptic AR-regulated enhancer in the SOX9 gene (24985976)[31], it was not surprising to find a strong link between the ERG-fusion positive genotype and strong SOX9 expression. The inclusion of deletion data from multiple chromosomal loci revealed further, that the relationship of SOX9 and ERG expression largely depended on whether or not a PTEN deletion was present in a tumor. Moreover, the data demonstrated a strikingly poor disease outcome in a subgroup of 101 patients with ERG positive, PTEN deleted cancers with absent SOX9 expression. These findings suggest strong and clinically relevant interactions between these proteins. A functional relationship of PTEN and SOX9 is indeed supported by two studies using prostate cancer mouse models [14,16]. These studies have suggested a cooperative role of SOX9 and PTEN loss for prostate tumor formation [14] by SOX9-dependent inhibition of the retinoblastoma (RB1) tumor suppressor [16] in order to bypass cellular senescence induced by PTEN loss [32]. Such a cooperative role fits well to our observation that SOX9 expression was linked to PTEN deletion at least in the subset of cancers lacking ERG fusion.

However, the inverse association between loss of SOX9 overexpression and PTEN deletion found in ERG-positive cancers indicates that very high SOX9 protein levels—as induced by ERG-fusion—might not per se provide a selection advantage to PTEN-deleted cancer cells. Moreover, since progression to high-grade disease was paralleled by a reduction of SOX9 expression in ERG-positive cancers, very strong SOX9 overexpression may even counteract tumor growth. This could be mechanistically explained through antagonistic effects in pathways governed by both ERG and SOX9. For example, while ERG activates canonical ß-catenin/WNT signaling [33,34], SOX9 contributes to switching between growth-promoting and differentiation-initiating consequences of this pathway [35,36]. It is, thus, tempting to speculate that strong SOX9 overexpression inhibits progression through forced differentiation, and that ERG-positive cancer cells consequently need to undergo adaptation steps to adjust SOX9 expression to a level that is compatible with tumor progression. Since the worst prognosis was found for PTEN-deleted and ERG-positive tumors that completely lacked any detectably SOX9 expression, SOX9 might even impair tumor growth in this specific molecular background. Such a crucial role of the molecular environment on the tumor-promoting or tumor-suppressive activity of SOX9 is also supported by cell line and xenograft experiments showing that SOX9 overexpression can lead to enhanced tumor growth and invasion in some cell lines [13] but to reduced tumorigenicity in others [37].

Remarkably, our data suggest that SOX9 may provide substantial additional prognostic information beyond PTEN deletion—one of the strongest known single prognosticators in prostate cancer, but at the same time also demonstrate that the value of SOX9 as a putative molecular marker is limited to the small subset of about 10–15% of prostate cancers harboring both ERG-fusion and PTEN deletion. SOX9 is thus an interesting example for possibly many more genes that may exert either a tumor promoting or a tumor suppressive action depending on the individual molecular scaffold resulting from specific combinations of genomic changes in prostate cancer cells [33,34,38]. Such observations challenge the concept of generally applicable multiparameter prognostic tests that generate a simple prognosis score with a similar impact on all cancers.

In summary, the results of our study demonstrate that SOX9 is expressed in a large fraction of prostate cancers, but has a variable prognostic impact depending of the molecular environment. The striking limitation of the prognostic impact of SOX9 loss to the subset of ERG-positive cancers with PTEN deletions provides further evidence for the existence of clinically relevant molecularly distinct subgroups of prostate cancers.

## Supporting Information

S1 Table Clinico-pathological association of SOX9 immunostaining in the ERG negative subset. (DOC)

S2 Table Multivariate analysis including SOX9 expression status in ERG-positive and PTEN deleted prostate cancers. (DOC)

S3 Table Numbers of patients at risk of biochemical recurrence in ERG-negative and ERG positive cancers. (XLS)
