# Supplementary material for: Loss of SOX9 Expression Is Associated with PSA Recurrence in ERG-Positive and PTEN Deleted Prostate Cancers
Source: PLoS One. 2015 Jun 1;10(6):e0128525. doi: 10.1371/journal.pone.0128525 (PMC4452277; doi:10.1371/journal.pone.0128525)
Supplement: S1 Table — (DOC) [file pone.0128525.s001.doc]

| **S1 Table. Clinico-pathological association of SOX9 immunostaining in the ERG negative subset.** | | | | | | | |
| --- | --- | --- | --- | --- | --- | --- | --- |
|  |  |  |  |  |  |  |  |
|  | **Evaluable (N)** | **SOX9 (%)** | | | | **P value** |  |
|  |  | **negative** | **weak** | **moderate** | **strong** |  |  |
| **Total** | 3,671 | 45 | 17 | 30 | 8 |  |  |
| **Tumor stage** | | | | | | | |
| pT2 | 2,454 | 45 | 16 | 30 | 9 | 0.001 |  |
| pT3a | 758 | 49 | 17 | 26 | 9 |  |  |
| pT3b | 427 | 38 | 20 | 37 | 6 |  |  |
| pT4 | 20 | 50 | 25 | 15 | 10 |  |  |
| **Gleason grade** | | | | | | | |
| ≤3+3 | 766 | 55 | 12 | 26 | 7 | <0.0001 |  |
| 3+4 | 2,094 | 42 | 18 | 31 | 9 |  |  |
| 4+3 | 603 | 42 | 19 | 32 | 7 |  |  |
| ≥4+4 | 191 | 41 | 20 | 27 | 11 |  |  |
| **Lymph node metastasis** | | | | | | | |
| N0 | 2,121 | 43 | 18 | 31 | 8 | 0.96 |  |
| N+ | 186 | 42 | 19 | 31 | 9 |  |  |
| **Preoperative PSA level (ng/ml)** | | | | | | | |
| <4 | 355 | 36 | 16 | 36 | 13 | <0.0001 |  |
| 4-10 | 2,202 | 44 | 17 | 31 | 9 |  |  |
| >10-20 | 811 | 46 | 19 | 28 | 7 |  |  |
| >20 | 272 | 57 | 15 | 22 | 6 |  |  |
| **Surgical margin** | | | | | | | |
| negative | 2,934 | 44 | 17 | 31 | 9 | 0.44 |  |
| positive | 672 | 48 | 16 | 28 | 8 |  |  |
