# Supplementary material for: Loss of SOX9 Expression Is Associated with PSA Recurrence in ERG-Positive and PTEN Deleted Prostate Cancers
Source: PLoS One. 2015 Jun 1;10(6):e0128525. doi: 10.1371/journal.pone.0128525 (PMC4452277; doi:10.1371/journal.pone.0128525)
Supplement: S2 Table — (DOC) [file pone.0128525.s002.doc]

**S2 Table. Multivariate analysis including SOX9 expression status in ERG-positive and PTEN deleted prostate cancers.**
